# Health and Economic Benefits of Routine Childhood Immunizations in the Era of the Vaccines for Children Program — United States, 1994–2023

**DOI:** 10.15585/mmwr.mm7331a2

**Published:** 2024-08-08

**Authors:** Fangjun Zhou, Tara C. Jatlaoui, Andrew J. Leidner, Rosalind J. Carter, Xiaoyu Dong, Jeanne M. Santoli, Shannon Stokley, Demetre C. Daskalakis, Georgina Peacock

**Affiliations:** 1 National Center for Immunization and Respiratory Diseases, CDC.

**Summary**

**What is already known about this topic?**

Broad access and availability of vaccines is critical for immunization programs to avert disease. Since 1994, the U.S. Vaccines for Children (VFC) program has covered the cost of vaccines for children whose families might not otherwise be able to afford them.

**What is added by this report?**

Among children born during 1994–2023, routine childhood vaccinations will have prevented approximately 508 million cases of illness, 32 million hospitalizations, and 1,129,000 deaths, resulting in direct savings of $540 billion and societal savings of $2.7 trillion.

**What are the implications for public health practice?**

During the VFC program era, routine childhood immunizations in the United States have been an important cost-saving public health strategy. Childhood immunizations continue to provide substantial health and economic benefits and promote health equity.

## Abstract

Since 1994, the U.S. Vaccines for Children (VFC) program has covered the cost of vaccines for children whose families might not otherwise be able to afford vaccines. This report assessed and quantified the health benefits and economic impact of routine U.S. childhood immunizations among both VFC-eligible and non–VFC-eligible children born during 1994–2023. Diphtheria and tetanus toxoids and acellular pertussis vaccine; *Haemophilus influenzae* type b conjugate vaccine; oral and inactivated poliovirus vaccines; measles, mumps, and rubella vaccine; hepatitis B vaccine; varicella vaccine; pneumococcal conjugate vaccine; hepatitis A vaccine; and rotavirus vaccine were included. Averted illnesses and deaths and associated costs over the lifetimes of 30 annual cohorts of children born during 1994–2023 were estimated using established economic models. Net savings were calculated from the payer and societal perspectives. Among approximately 117 million children born during 1994–2023, routine childhood vaccinations will have prevented approximately 508 million lifetime cases of illness, 32 million hospitalizations, and 1,129,000 deaths, at a net savings of $540 billion in direct costs and $2.7 trillion in societal costs. From both payer and societal perspectives, routine childhood vaccinations among children born during 1994–2023 resulted in substantial cost savings. Childhood immunizations continue to provide substantial health and economic benefits, while promoting health equity.

## Introduction

Immunizations have contributed to substantial declines in morbidity and mortality associated with vaccine-preventable diseases worldwide. Broad availability of and access to vaccines is critical to averting disease and maximizing health benefits. In response to a U.S. measles resurgence during 1989–1991, the U.S. Congress established the Vaccines for Children (VFC) program in 1994 to provide vaccines at no cost to eligible children (*1*). Children can receive vaccines through VFC if they are Medicaid-eligible, uninsured, underinsured,* or American Indian or Alaska Native (*2*). In 2023, approximately 54% of children aged ≤18 years were eligible to receive VFC vaccines (CDC, unpublished data, 2023).

VFC has provided vaccines targeting nine diseases for eligible children aged ≤6 years since the program began in 1994: diphtheria, tetanus, and pertussis (DTP, [later, acellular pertussis, DTaP]) vaccine; *Haemophilus influenzae* type b (Hib) vaccine; polio (oral poliovirus vaccine [OPV] then inactivated [injectable] poliovirus vaccine [IPV]); measles, mumps, and rubella (MMR) vaccine; and hepatitis B (HepB) vaccine. Vaccines or immunizing agents targeting seven additional diseases were added to the routine immunization schedule for children aged ≤6 years^†^ during 1996–2023: varicella vaccine (VAR; 1996); hepatitis A vaccine (HepA; 1996–1999 for high-risk areas and 2006 for all states); pneumococcal conjugate vaccine (PCV) (7-valent [PCV-7] in 2000, 13-valent [PCV-13] in 2010, 15-valent [PCV-15] in 2022, and 20-valent [PCV-20] in 2023); influenza (for children aged 6–23 months in 2004 and for those aged 6–59 months in 2006); rotavirus vaccine (Rota; 2006); COVID-19 vaccine (2023); and respiratory syncytial virus vaccine (RSV; 2023). This report summarizes the health benefits and economic effects of routine U.S. childhood immunization among all children (both VFC- and non–VFC-eligible) born during 1994–2023.

## Methods

### Vaccines Included in Analysis

Following previously established methods (*2*,*3*), one decision tree for each vaccine was used as the basis for the models, and the effects of routine childhood vaccination with nine vaccines (DTP/DTaP, Hib, OPV/IPV, MMR, HepB, VAR, HepA, PCV, and Rota) on 30 annual cohorts of children born during 1994–2023 were evaluated.^§^ Although influenza and COVID-19 vaccines are recommended for routine immunization, they were not included in this analysis, because the methods for assessing their costs and effects differ from those for other vaccines. In addition, recently recommended RSV vaccines were also not included, because implementation had just commenced in 2023, and some product supplies were constrained.

### Data Sources

Immunization program costs, costs of disease outcomes, and parent travel and work time lost were estimated from birth through death (based on life expectancy^¶^) for the children in each birth cohort (*3*). Vaccination coverage with each of these vaccines in the United States during 1994–2022, estimated by the National Immunization Surveys (NIS) (*4*) and school vaccination surveys, were used (*5*). Data from 2022 were used to estimate the benefits and costs for 2023 because most of 2023 data were not available.

The age-specific annual incidences of diphtheria, tetanus, pertussis, Hib, poliomyelitis, measles, mumps, rubella, varicella, hepatitis A, rotavirus, and pneumococcus-related diseases, and the prevalence, complications, and perinatal transmission of hepatitis B in the United States in the prevaccine era were obtained from a previous analysis and used to estimate the morbidity and mortality of disease during that period (*3*). Disease morbidity and mortality after vaccination were estimated using surveillance data from the National Notifiable Diseases Surveillance System,** West Philadelphia Varicella Active Surveillance Project, and the Active Bacterial Core Surveillance.^††^ For three vaccines (HepB, HepA, and Rota), the estimates for disease morbidity and mortality were model-based using vaccination coverage and efficacy (*3*).

### Outcomes Estimated and Calculations

Net savings and benefit-cost ratios for all nine vaccines were calculated. Benefits of routine childhood immunization were quantified as the savings in direct and indirect costs from averting morbidity and mortality by vaccination. The immunization program costs are jointly covered by parents and private and public sectors, including VFC, and estimated using data from the CDC Vaccine Price List^§§^ and from a previous analysis (*3*). These costs comprise the vaccines, administration, parent travel and work time lost, and adverse events associated with receipt of these vaccines. Net savings is the sum of the benefits from routine childhood immunization with the nine vaccines minus the sum of the immunization program costs, and benefit-cost ratio was calculated as the benefits divided by the immunization program costs. To account for the differential timing of benefits and costs, all future benefits and costs were discounted annually at 3%, as recommended by the Second Panel on Cost-Effectiveness in Health and Medicine (*6*).

The analyses were performed from two perspectives: payer (direct medical and nonmedical costs) and societal (direct and indirect costs). Direct medical costs include those associated with treating an initial infection, as well as the lifetime costs associated with complications and sequelae of these vaccine-preventable diseases. Direct nonmedical costs include those for travel, special education of children disabled by specific vaccine-preventable diseases, and disability-related supplies. Indirect costs include productivity losses attributable to premature mortality and permanent disability among cohort members, as well as opportunity costs associated with parents who miss work to care for their sick children or cohort members themselves who miss work because of vaccine-preventable illness. All costs were adjusted to the 2023 U.S. dollar. The general Consumer Price Index was used for productivity losses, opportunity and travel costs, and the medical care component of the Consumer Price Index was used for direct medical costs.^¶¶^ This activity was reviewed by CDC, deemed not research, and was conducted consistent with applicable federal law and CDC policy.***

## Results

### Vaccination-Associated Prevention of Morbidity and Mortality

Among approximately 117 million children born during 1994–2023, routine childhood immunization was estimated to prevent 508 million lifetime cases of illness (averaging four illnesses per child) and 32 million hospitalizations (0.3 per child) and to avert 1,129,000 premature deaths from vaccine-preventable illnesses (Table 1). The cumulative number of illnesses prevented ranged from 5,000 for tetanus to approximately 100 million for measles and varicella. The highest estimated cumulative number of hospitalizations and deaths prevented were 13.2 million hospitalizations for measles vaccination and 752,800 deaths for diphtheria vaccination.

**TABLE 1:** Estimated number of illnesses, hospitalizations, and deaths prevented by routine childhood immunization against selected vaccine-preventable diseases in 30 cohorts of children — United States, 1994–2023

Vaccine-preventable disease	Illnesses prevented (x 1,000)	Hospitalizations prevented (x 1,000)	Deaths prevented (x 1,000)
Diphtheria	7,528	7,528	752.8
Tetanus	5	5	0.7
Pertussis	80,738	3,646	28.4
*Haemophilus influenzae* type b	536	495	20.3
Polio	1,847	786	21.9
Measles	104,984	13,172	85.0
Mumps	63,355	2,020	0.3
Rubella	54,225	199	0.4
Congenital rubella syndrome	17	26	1.9
Hepatitis B	6,061	940	90.1
Varicella*	106,270	272	1.9
Hepatitis A*	4,048	78	1.5
Pneumococcus-related diseases*^,†^	47,804	1,969	123.2
Rotavirus*	30,265	819	0.4
**Total**	**507,683**	**31,955**	**1,128.8**

* Varicella vaccine for 1996–2023 cohorts, hepatitis A vaccine for 2006–2023, pneumococcal conjugate vaccine for 2001–2023, and rotavirus vaccine for 2007–2023.

^†^ Includes invasive pneumococcal disease, otitis media, and pneumonia.

### Economic Effect of Vaccination

Vaccination for the 1994–2023 birth cohorts will potentially avert $780 billion in direct costs and $2.9 trillion in societal costs by preventing illnesses and deaths (Table 2). After accounting for $240 billion in direct costs and $268 billion in societal costs of routine childhood immunization, the net savings for routine childhood immunization from the payer and societal perspectives were $540 billion and $2.7 trillion, respectively. The payer and societal benefit-cost ratios for routine childhood immunizations were 3.3 and 10.9, respectively.

**TABLE 2:** Lifetime health and economic outcomes in 30 cohorts of children — United States, 1994–2023

Outcome	All children born 1994–2023
**Total illnesses prevented (x 1,000)**	**507,683**
**Total hospitalizations prevented (x 1,000)**	**31,955**
**Total deaths prevented (x 1,000)**	**1,129**
Direct cost of immunization (billion, $)	240
Societal cost of immunization (billion, $)	268
Benefits in direct costs (billion, $)	780
Benefits in societal costs (billion, $)	2,931
Direct net savings (billion, $)	540
Societal net savings (billion, $)	2,663
Payer benefit-cost ratio*	3.3
Societal benefit-cost ratio^†^	10.9

* Payer benefit-cost ratio = benefits in direct costs / direct cost of immunization.

^†^ Societal benefit-cost ratio = benefits in societal costs / societal cost of immunization.

## Discussion

Routine childhood immunizations remain a highly cost-effective public health intervention, preventing thousands of lifetime illnesses, hospitalizations, and deaths among children born during 1994–2023. Based on the 2022 CDC Market Share Report (CDC, unpublished data, 2022 [2023 data are not available]), VFC made a substantial contribution to these reductions by purchasing approximately one half of childhood vaccines at discounted prices.

Accurately estimating the proportion of benefits attributable to VFC is challenging because a child's eligibility for the VFC program can change over time. In addition, the percentage of vaccines purchased by VFC varies each year and by vaccine type. These variations complicate consistent measurement of the program’s direct effect on public health outcomes. Coverage with many of the vaccines included in this analysis was near or above 90% during 1994–2022 (2023 NIS data were not available) (*4*,*5*). The VFC program reduces financial and logistical barriers for eligible children who otherwise might not have reasonable access to immunization, thereby promoting health equity and contributing substantially to these high coverage levels. Whereas the societal costs of routine childhood immunization over 30 cohorts of children are $268 billion, the resulting societal savings are $2.9 trillion. This calculation means that every $1 spent on childhood immunizations results in a savings of approximately $11. With discounted vaccine prices, every $1 spent on VFC program results in even further savings.

VFC funds are allocated to CDC by the Centers for Medicare & Medicaid Services, and Medicaid providers can receive payment from Medicaid for vaccine administration services provided to Medicaid-eligible children.^†††^ CDC provides funding to 61 state, local, and territorial immunization programs to implement and oversee the VFC program and relies on participation from public and private health care providers to administer vaccines to eligible children.

During the COVID-19 pandemic, routine childhood vaccination coverage declined, in part resulting from reduced primary care service availability and increases in vaccine hesitancy (*7*). During the same period, the spread of vaccine-related misinformation and disinformation affected vaccine confidence (*8*) and threatened high vaccination coverage rates. Recent measles outbreaks resulting from internationally imported measles cases serve as a reminder that high vaccination coverage is critical for protection from highly transmissible vaccine-preventable diseases (*9*). VFC plays an important role in maintaining high childhood vaccination coverage by reducing barriers to access, especially in geographic areas and among populations that have historically had lower vaccination coverage, such as children living in rural areas (*4*). The VFC program is one of the nation’s primary health platforms for promoting health equity, and VFC providers are critical to facilitating equitable vaccine access. Immunization programs might consider expanding their provider network by using nontraditional vaccine providers such as pharmacies in areas where access is deemed to be inadequate. Further, provider reminders, provider assessment and feedback, and client reminder-recall systems remain important methods to reduce missed opportunities for vaccination. VFC also serves as a critical component of U.S. preparedness by supporting important infrastructure needed for distributing medical countermeasures to children to halt transmission of vaccine preventable disease or mitigate severity of illness in outbreak settings.

### Limitations

The findings in this report are subject to at least four limitations. First, influenza, COVID-19, and RSV immunization were not included in this analysis, which might result in an underestimate of the benefits attributable to the immunization program. Second, actual vaccination coverage with some vaccines might be higher than estimates provided by NIS surveys (*10*), which might result in underestimating immunization costs. Third, federal, state, and local immunization program management expenditures and excise taxes^§§§^ were not included, which might also result in underestimating immunization costs. Finally, for some diseases, factors other than immunization (e.g., hygiene and physical distancing measures) might have contributed to lower disease risks in recent decades, and reductions resulting from these contributions have not been incorporated into the model. If such reductions were substantial, the model would overestimate the vaccine-preventable incidence of these diseases. However, a sensitivity analysis from a previous study found that even with worst-case scenario assumptions, routine childhood immunization remained cost-saving (*3*). As noted, estimating more precise effects of vaccinations among children enrolled in the VFC program involves several complexities outside the scope of this report.

### Implications for Public Health Practice

Supported by the VFC program, immunization has been a highly effective tool for improving the health of U.S. children. This analysis demonstrates the continued and substantial health benefits associated with vaccinating young children, rendering the investment in vaccines and immunizations services an important and cost-saving public health strategy.

## Footnotes

* Children categorized as underinsured because their health plans do not include coverage for recommended vaccinations are eligible to receive VFC vaccines only at Federally Qualified Health Centers, Rural Health Clinics, or under an approved deputization provider location agreement. https://www.cdc.gov/vaccines-for-children/hcp/program-eligibility/index.html

†
https://www.cdc.gov/vaccines/schedules/hcp/imz/child-adolescent.html

§ Population estimates from 1994–2022 at age 0: https://www.census.gov/programs-surveys/popest.html. Data from 2022 were used to estimate the costs and benefits for 2023. Range = 3,570,131–4,147,947 children per birth cohort.

¶
https://www.cdc.gov/nchs/nvss/life-expectancy.htm

**
https://www.cdc.gov/nndss/index.html

††
https://www.cdc.gov/abcs/index.html

§§ Discounted by 14%–72% for the vaccines included in this analysis. https://www.cdc.gov/vaccines-for-children/php/awardees/current-cdc-vaccine-price-list.html

¶¶
https://www.bls.gov/cpi/data.htm

*** 45 C.F.R. part 46.102(l)(2), 21 C.F.R. part 56; 42 U.S.C. Sect. 241(d); 5 U.S.C. Sect. 552a; 44 U.S.C. Sect. 3501 et seq.

††† Program for distribution of pediatric vaccines, Sec. 1928. [42 U.S.C. 1396s]. https://www.ssa.gov/OP_Home/ssact/title19/1928.htm

§§§ A $0.75 excise tax imposed on each component of (i.e., each disease prevented by) a vaccine is collected to fund the National Vaccine Injury Compensation Program. https://www.hrsa.gov/vaccine-compensation
